# Towards retrospective motion correction and reconstruction for clinical 3D brain MRI protocols with a reference contrast

**DOI:** 10.1007/s10334-024-01161-y

**Published:** 2024-05-17

**Authors:** Gabrio Rizzuti, Tim Schakel, Niek R. F. Huttinga, Jan Willem Dankbaar, Tristan van Leeuwen, Alessandro Sbrizzi

**Affiliations:** 1 Utrecht University, Heidelberglaan 8, 3584 CS Utrecht, The Netherlands; 2 Universitair Medisch Centrum Utrecht, Heidelberglaan 100, 3584 CX Utrecht, The Netherlands; 3 Centrum Wiskunde & Informatica, Science Park Amsterdam 123, 1098 XG Amsterdam, The Netherlands

**Keywords:** Brain imaging, Motion correction, Three-dimensional imaging

## Abstract

**Object:**

In a typical MR session, several contrasts are acquired. Due to the sequential nature of the data acquisition process, the patient may experience some discomfort at some point during the session, and start moving. Hence, it is quite common to have MR sessions where some contrasts are well-resolved, while other contrasts exhibit motion artifacts. Instead of repeating the scans that are corrupted by motion, we introduce a reference-guided retrospective motion correction scheme that takes advantage of the motion-free scans, based on a generalized rigid registration routine.

**Materials and methods:**

We focus on various existing clinical 3D brain protocols at 1.5 Tesla MRI based on Cartesian sampling. Controlled experiments with three healthy volunteers and three levels of motion are performed.

**Results:**

Radiological inspection confirms that the proposed method consistently ameliorates the corrupted scans. Furthermore, for the set of specific motion tests performed in this study, the quality indexes based on PSNR and SSIM shows only a modest decrease in correction quality as a function of motion complexity.

**Discussion:**

While the results on controlled experiments are positive, future applications to patient data will ultimately clarify whether the proposed correction scheme satisfies the radiological requirements.

**Supplementary Information:**

The online version contains supplementary material available at 10.1007/s10334-024-01161-y.

## Introduction

Magnetic resonance imaging (MRI) is fundamentally prone to motion artifacts, since the data acquisition process usually lasts several minutes for each acquired contrast, and the MR exam can be an uncomfortable experience for the patient. Motion corruption impedes a correct radiological assessment, which then may require a scan repetition, leading to considerable waste of resources for the hospital [1].

Motion reduction strategies are broadly classified as preventive, prospective, or retrospective techniques [2, 3]. Preventive strategies include physical devices to limit the motion (e.g., head holders) or sedation, but their application is limited by ethical or health considerations, and are often ineffective in eliminating patient movement. Prospective and retrospective strategies, on the other hand, directly or indirectly estimate the motion that the object of interest undergoes inside the scanner, and remove its effect from the data or in the reconstruction phase. This correction step is said to be applied “prospectively” [4] when the position of the patient is tracked in real time and the scan settings are adjusted accordingly on-the-fly. For example, the relative change of position can be estimated by acquiring additional *k*-space or image-space navigators [5, 6], or with “self-navigating” sequences [7–9]. Alternatively, camera devices or markers [10, 11] can be used to estimate the imaging object position. However, most tracking modalities are often defective in terms of either precision, patient interaction, or sequence independence [4]. Therefore, although effective in many respects, prospective methods have somewhat limited range of application.

Retrospective algorithms are characterized by the removal of motion artifacts in the final reconstruction phase, after the data acquisition. The main advantage of retrospective schemes is in their flexibility, since they do not necessarily require additional hardware, scanner modifications, MR navigators, markers, and so on. Note, however, that they may benefit from using prior information about the target imaging object and motion pattern. One main challenge for this class of methods is the need for time-intensive computations. The scientific literature on retrospective motion correction is quite rich: examples of retrospective techniques for rigid motion using navigators or markers can be found in [5, 9, 12–14], while examples of “blind” techniques (in this context, meaning that they are not using navigators or markers) are presented in [15–19].

Retrospective correction schemes are typically formulated as a bi-level optimization problem, where two types of unknowns are jointly estimated: the reconstructed (2D/3D) image and the motion parameters. Due to the ill-posedness of the problem here considered, the choice of the regularization method is crucial: see, for example, gradient-entropy regularization in [17–19], sparsity regularization in [20], or iteratively re-weighted least-squares regularization in [21]. Another strategy to ease the ill-posedness is to resort to special acquisition patterns in *k*-space that are more robust in terms of motion correction, as described in the DISORDER method in [21]. Alternatively, many machine-learning approaches have been recently proposed for retrospective motion correction [22–28].

Some previous work in [29] introduced a retrospective motion correction scheme, whose novel aspect is the use of a contrast free of motion artifacts that can be leveraged as a reference to remove motion effects from any other contrast from the same patient, akin to a generalized rigid motion registration. The chief assumption of this work is the following: in a multi-contrast MR session, motion does not typically affect all the scans and some motion-free scans are generally available, so that we can exploit their anatomic similarity. Structural similarity is technically achieved via structure-guided total variation (TV), as originally proposed in [30] and further developed in [31] (see also [32]).

The goal of this paper is to extend the scope of [29], limited to 2D synthetic results, to general 3D randomized acquisitions and 3D rigid-motion correction. We experimentally verify that a 3D extension is indeed feasible for brain imaging. We do not assume data-driven priors (so that machine learning is not available), any additional navigator data, nor consider motion-resilient acquisition schemes, to conform to more broadly available clinical protocols. Note that the proposed method can employ any acquisition scheme, in principle, but we stick to Cartesian acquisition, which are the standard encoding strategies of clinical protocols. Since we focus on brain imaging, rigid motion can be effectively assumed for our scope. The reference and the corrupted contrast do not need be co-registered or acquired with the same resolution.

We thoroughly validate the method with a prospective in-vivo study based on three volunteers and several motion types. The strength and limitations of the method are highlighted with the comparison of correction quality with varying degrees of motion artifacts and contrast type as a reference prior.

## Theory

In this section, we present the basic mathematical formulation underpinning the proposed motion correction method (further details can be found in [29]). The contrast volume, in the remainder of this section, will be denoted by ${{\mathbf{u}}} \in {{\mathbb{C}}}^{n_x }$, where $n_x$ is the number of voxels contained in a rectangular field of view. The 3D image undergoes a time-dependent rigid motion

1 $${\mathbf{u}}_t = T_{\theta_t } {\mathbf{u}},$$

where $t$ is a time-related label. In practice, $t$ corresponds to the index of the *k*-space readout line in the phase-encoding plane. The corresponding rigid transformation is given by $T_{{{\varvec{\theta}}}_t }$, and is parameterized by a time-dependent motion parameter ${{\varvec{\theta}}}_t \in {{\mathbb{R}}}^6$, which includes translations and rotations in 3D:

2 $${{\varvec{\theta}}} = \left( {{{\varvec{\tau}}} ,{\varvec{\varphi }}} \right),\quad {{\varvec{\tau}}} = \left( {\tau_x , \tau_y , \tau_z } \right),\quad {\varvec{\varphi }} = \left( {\varphi_x , \varphi_y , \varphi_z } \right).$$

The rigid motion consists of a 3D rotation (defined by the 2D rotation angles $\varphi_x , \varphi_y , \varphi_z$, performed in this order around the corresponding axes) followed by a translation (governed by the translation parameters $\tau_x , \tau_y , \tau_z$.

Without loss of generality, we are assuming a Cartesian acquisition. At each given time $t$, the MR acquisition process corresponds to the evaluation of the Fourier transform ${{\mathcal{F}}}$ of $u_t$ in a particular subset $K_t$ of the *k*-space. In practice, the acquisition is structured in such a way that all the subsets $K_t$ consist of parallel lines in the *k*-space (the common direction being the readout direction). We refer to the Fourier transform of a rigidly moving object $u_{{\varvec{\theta}}} : = T_{{\varvec{\theta}}} u$ as the perturbed Fourier transform ${{\mathcal{F}}}_{{\varvec{\theta}}} u: = {{\mathcal{F}}}u_{{\varvec{\theta}}}$, and can be directly characterized as

3 $${{\mathcal{F}}}_{{\varvec{\theta}}} u \left( {{\mathbf{k}}} \right): = {\text{exp }}\left( { - {\text{i }}k \cdot { }\tau } \right){ }{{\mathcal{F}}}u \left( {R_{\varvec{\varphi }}^{ - 1} u} \right)$$

where the rotational operator with respect to the 3D angle ***φ*** is indicated by $R_{\varvec{\varphi }}^{\,}$. This definition is motivated by classical Fourier identities that describe the action of rigid motion under the Fourier transform. Due to rotational effects, one must resort to the non-uniform discrete Fourier transform (NUFFT) to evaluate Eq. (3) [33, 34].

Note that we implicitly assumed that no motion occurs while sampling the elements of $K_t$, since the state of the object at the time $t$ is associated to a single motion parameter ${{\varvec{\theta}}}_t$. The assumption is motivated by the fact that $K_t$ will correspond, in practice, to a single Cartesian readout line, which lasts few milliseconds. Hence, the data acquisition at time $t$ is symbolized by the application of the selection operator $S_t$ to the Fourier-transformed volume:

4 $$d_t = { }S_t { }{{\mathcal{F}}}_{{{\varvec{\theta}}}_t } \mathbf{u}{ } = { }\left( {{{\mathcal{F}}}_{{{\varvec{\theta}}}_t } \mathbf{u}{ }\left( {\mathbf{k}_1 } \right),{ }.{ }.{ }.{ },{{\mathcal{F}}}_{{{\varvec{\theta}}}_t } \mathbf{u}{ }\left( {\mathbf{k}_{n_r } } \right)} \right),\quad \mathbf{k}_1 ,{ }.{ }.{ }.{ },{ }\mathbf{k}_{n_r } \in K_t .$$

Here, $n_r$ is the number of *k*-space samples in a single readout. The resulting inverse problem can be cast as an optimization problem over the reconstruction unknowns **u** and the motion parameters ${{\varvec{\theta}}}_t$, that is:

5 $${\mathop {\min }\limits_{\mathbf{u}, {{\varvec{\theta}}}_{1:n_t } }} f\left( {\mathbf{u}, {{\varvec{\theta}}}_{1:n_t } } \right) + \mu\, g_\theta \left( { {{\varvec{\theta}}}_{1:n_t } } \right)\quad {\text{s}}{\text{.t}}.\quad g_u \left( \mathbf{u} \right) \le \varepsilon$$

where ${{\varvec{\theta}}}_{1:n_t } = ( {{\varvec{\theta}}}_1 , \ldots , {{\varvec{\theta}}}_{n_t } )$, and $n_t$ is the number of time steps. The parameters $\varepsilon , \mu$ (both positive numbers) set the strength of the corresponding regularization terms. The first term of the objective functional in Eq. (5) corresponds to the data misfit:

6 $$f\left( {\mathbf{u}, {{\varvec{\theta}}}_{1:n_t } } \right) = \mathop \sum \limits_{t = 1}^{n_t } \frac{1}{2}\|{{\mathcal{F}}}_{{{\varvec{\theta}}}_t } {\mathbf{u }} - \mathbf{d}_t \|^2 .$$

The least-squares norm is indicated here by ∥·∥. The regularization terms $g_u$ and $g_\theta$ are crucial in ensuring the well-posedness of the problem. Indeed, the objective in Eq. (6) will be sensitive to the relatively low signal-to-noise ratios of the high-frequency components of the data. Moreover, the objective is highly non-convex as a function of ${{\varvec{\theta}}}_{1:n_t }$. The motion-parameter regularization is designed to ensure some form of regularity in time (e.g., smoothness), this can be achieved for example by setting

7 $$g_\theta \left( {{{\varvec{\theta}}}_{1:n_t } } \right) = \mathop \sum \limits_{t = 1}^{n_t - 1} \frac{1}{2}\|{{\varvec{\theta}}}_{t + 1} - {{\varvec{\theta}}}_t \|^2 .$$

Alternatively, higher-order derivatives may be used. Another strategy, adopted in this paper, is to impose smoothness by setting hard constraints for the motion parameters, rather than via an additive penalty term as in Eq. (7) [29].

### Reference-guided total variation regularization

The crux of the proposed method is related to the choice of the regularization term $g_u$ in Eq. (5). We adopt the structure-guided total variation scheme proposed in [30] in the context of multi-contrast imaging, that is:

8 $$g_u \left( \mathbf{u} \right) = \sum \limits_{\mathbf{x}}\| {\Pi }_{\mathbf{v}} |_{\mathbf{x}} \nabla \mathbf{u}|_{\mathbf{x}}\| ,\quad\quad {\Pi }_{\mathbf{v}} |_{\mathbf{x}} = I_3 - {\upxi }_{\mathbf{v}} |_{\mathbf{x}} \upxi_{\mathbf{v}} |_{\mathbf{x}}^{\text{H}} ,$$

where $I_3$ is the 3 × 3 identity matrix, $\nabla \cdot |_{\mathbf{x}}$ is the discretized gradient operator evaluated at the voxel with center **x**, and ${\Pi }_{\mathbf{v}} |_{\mathbf{x}}$ is the projection operator on the linear space that is orthogonal to the vector ${\upxi }_{\mathbf{v}} |_{\mathbf{x}} \in {{\mathbb{C}}}^3$. The symbol ^H^ indicates the adjoint operation. The vector ${\upxi }_{\mathbf{v}} |_{\mathbf{x}}$ corresponds to the normalized gradient of a given motion-free contrast **v**, e.g., $\upxi_{\mathbf{v}} |_{\mathbf{x}} \approx \nabla \mathbf{v}|_{\mathbf{x}} / \|\nabla \mathbf{v}|_{\mathbf{x}}\|$. The actual definition is

9 $${\upxi }_{\mathbf{v}} |_{\mathbf{x}} = \frac{\nabla \mathbf{v}|_{\mathbf{x}} }{{\sqrt {\|\nabla \mathbf{v}|_{\mathbf{x}}\|^2 + \eta^2 } }} ,$$

for some constant *η* > 0. The regularization term in Eq. (8) enforces the gradient structure of **v** onto **u**, when **v** and **u** are anatomically compatible. It is important to observe that **v** is not required to be registered with the target contrast $\mathbf{u}$, since the estimation of the motion parameters in Eq. (5) will automatically compensate for the initial misalignment (see also [31]). In this work, we actually adopt a constrained formulation, meaning that structural similarity is imposed by forcing the solution to belong to the constraint set $C_u = \{ \mathbf{u} : g_u \left( \mathbf{u} \right) \le \varepsilon \}$, where $\varepsilon > 0$ is a prescribed regularization level (see [29, 35], for more details).

### Optimization

To solve Eq. (5), we adopt an alternating update scheme based on the proximal alternating minimization algorithm (PALM) described in [36]. The algorithm of the optimization strategy is exemplified in Algorithm 1. Each update requires the linearization of the smooth objective $f$ and the application of the proximal operators associated to $g_u$ and $g_\theta$. We will make use of multi-scale methods to ease the ill-posedness of the problem. Two types of scale are considered, here:

- spatial/temporal grid: this scale is associated to the spatial and temporal grid sizes of the reconstructed image ${{\mathbf{u}}}$ and motion parameters ${{\varvec{\theta}}}_{1:n_t }$, respectively, by considering a sequence of optimization problems defined on progressively finer grids. The pragmatic approach considered in this work actually consists in fixing a relatively coarse temporal grid for the motion unknowns, along with a corresponding time-interpolation operator (this procedure effectively acts as an additional implicit regularizer for ${{\varvec{\theta}}}_{1:n_t }$). Therefore, only the spatial grid of ${{\mathbf{u}}}$ is scaled up. Note that the spatial scale considered at a certain multi-scale stage poses a limit on how well the motion parameters can be resolved at that stage, due to the Nyquist criterion, since they are associated to specific coordinates in *k*-space;
- regularization strength: this scale is related to the regularization level $\varepsilon$, as defined in Eq. (5). Hence, strongly regularized problems are solved first, and the regularization is gradually relaxed as in a continuation strategy. As mentioned above, this regularization term is explicitly implemented by forcing the solution to lay in the set $C_u = \{ \mathbf{u} : g_u \left( \mathbf{u} \right) \le \varepsilon \}$, where $\varepsilon > 0$ and $\varepsilon = \alpha\, g_u \left( {\mathbf{u}_{{\text{corrupted}}} } \right)$ and $\alpha = 0.01, 0.1, 0.5, 0.8$. This choice was preliminarily fine-tuned on earlier results. See for more details [29].

Overall, this results in two nested sequences of optimization problems (see Algorithm 1).

**Figure:**
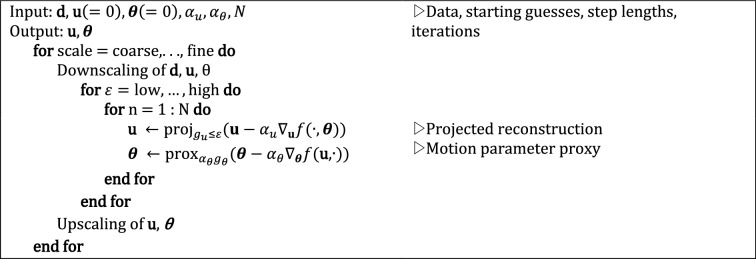
**Algorithm 1** Joint motion correction and reconstruction with alternating proximal operator evaluation

### Rigid motion parameter conventions

The proposed motion correction algorithm estimates the rigid motion ${{\varvec{\theta}}} = ({{\varvec{\tau}}} ,{\varvec{\varphi }})$ that the object of interest undergoes at some point during the scan, to undo its effect on the reconstructed 3D image. We parameterize the rigid motion in terms of three translation parameters ${{\varvec{\tau}}} = (\tau_x , \tau_y ,\tau_z )$, each one corresponding to motion in the spatial coordinate direction $x, y, {\text{or}} z,$ and three rotation angles ${\varvec{\varphi }} = \left( {\varphi_x , \varphi_y , \varphi_z } \right)$, which describe 2D rotations around the axes $x, y, z$, respectively. We conventionally assume that the motion is performed by an initial translation, followed by three plane rotations. The order of these rotations is implicitly defined by ${\varvec{\varphi }}$.

For a consistent display of our results, we are assuming that: the $x$ direction corresponds to the left–right direction, $y$ to the posterior-anterior direction, and $z$ to the inferior-superior direction, the $xy$ plane corresponds to the axial plane, $xz$ to the coronal plane, and $yz$ to the sagittal plane. Left/right, anterior/posterior, and inferior/superior are meant from the patient perspective. The orientation of the rotation planes is determined by the lexicographic order of their labels, according to the right-hand rule.

## Methods

In this section, we set up several experiments that demonstrate the capabilities of the retrospective motion correction algorithm detailed in “Theory” section, whose main novel aspect and strength is the use of a reference contrast to guide the correction. Our objective is to tackle motion correction for brain imaging, and we focus on acquisition protocols that are relevant for the clinical practice.

All the imaging sequences considered in this study were taken from actual clinical brain protocols of the Radiology and Radiotherapy departments of the UMC Utrecht. The data considered in this section is based on 3D Cartesian acquisition. The sampling pattern used in these acquisitions typically utilizes pseudo-random undersampling. The main assumptions underlying the proposed method are related to the availability of a motion-free reference contrast and the motion artifacts being produced by rigid motion.

We consider several studies with volunteer data (three volunteers in total^1^), where motion artifacts are prospectively generated by instructing the volunteer to actively move during the scan (a certain number of times). While we did not track the type of rigid motion produced by the volunteers, we prompted them to maintain the same position in between our instructions. In this way, we have some fair qualitative expectations about the motion estimated by the correction algorithm (that is, a stepwise behavior). The ‘ground-truth’ acquisition and reconstruction is obtained by simply asking the volunteers not to move. Note that tests were not repeated for reproducibility.

The volunteer studies aim at investigating several relevant questions related to the application of the proposed retrospective motion correction technique. The first study in “Experiment 1: Robustness with respect to motion complexity” section is a qualitative assessment of the robustness of the motion correction with respect to motion complexity, here equated to the number of volunteer poses during the scan. In “Experiment 2: On the choice of the reference contrast” section, we demonstrate that many combinations of corrupted-contrast and reference-contrast types are possible for adequate correction. In the experiment in “Experiment 3: Scanner reconstruction versus processed raw *k*-space data as input for retrospective motion correction” section (and, additionally, in Appendix B, online resource 1), we ascertain under which conditions using the reconstructed complex DICOM image extracted from the scanner (which comprises both amplitude and phase), followed by a Fourier transform, is suited as input *k*-space data for the proposed motion-correction Algorithm 1. We note that the proposed method assumes coil-combined data as input for computational reasons, therefore it is sensitive to the way the raw *k*-space data is post-processed, and, in particular, to the degree of which the post-processed data can be adequately corrected by rigid-motion estimation. Finally, further experimentation is deferred to the supplemental section in Appendix C (online resource 1), where we demonstrate the effectiveness of the reference-based motion correction against a “blind” motion correction method, which does not use a reference contrast to eliminate the motion artifacts.

To further clarify the terminology “corrupted”, “corrected”, and “ground-truth”, referenced by the compared images throughout “Results” section, we report here a brief summary of the processing involved in obtaining such images:

- *ground-truth*: images obtained from the SENSE reconstruction of (coil-dependent) motion-free raw *k*-space data. The reconstruction process employs weak Tikhonov regularization weighting;
- *corrupted*: same processing for ground-truth images, except that the raw *k*-space data are affected by motion;
- *corrected*: these images are the output of the proposed motion-correction scheme detailed in “Theory” section. In this case, the input “data” for the algorithm (e.g., $d$ in Algorithm 1) consist in the array obtained by applying the Fourier transform to the corrupted image (previously described), and followed by a restriction to the *k*-space wavenumbers sampled by the original acquisition sequence.

All the following investigations use a 1.5 T Philips Ingenia scanner with a 15-channel head coil. We considered several contrast acquisition sequences with the specifications highlighted in Table 1. For all the experiments except the one described in “Experiment 3: Scanner reconstruction versus processed raw *k*-space data as input for retrospective motion correction” section, the raw *k*-space data (pertaining to corrupted or ground-truth scans) were exported for off-line processing.

**Table 1:** Specification of the acquisition sequences utilized in the experiments in “Methods” section

Exp.	Contrast	Sequence	Resolution (mm^3^)	FOV (mm^3^)	TR (ms)	TE (ms)	Flip angle (°)	Phase-encoding pattern	Duration (s)
“Experiment 1: Robustness with respect to motion complexity” section	T2-FLAIR	3D TSE	1.2	230 × 230 × 237.6	4800	320	90	Randomized	350
	T1(*)	3D TFE	1	230 × 230 × 238	7.8	3.6	8	Randomized	180
“Experiment 2: On the choice of the reference contrast” section	T1	3D TFE	1	230 × 230 × 238	7.9	3.6	8	Randomized	180
	T2(*)	3D TSE	1.1	250 × 250 × 190.3	3000	260	90	Randomized	180
“Experiment 3: Scanner reconstruction versus processed raw *k*-space data as input for retrospective motion correction” section	T2	3D TSE	1.3	250 × 250 × 183.26	2000	318	90	Regular (acc. 2 × 2)	300
	T1(*)	3D TFE	1	250 × 250 × 183	7.7	3.6	8	Randomized	150
“Experiment 3: scanner reconstruction versus processed raw *k*-space data as input for retrospective motion correction” section	T2-FLAIR	3D TSE	1.2	230 × 230 × 238	4800	291	90	Randomized	350
	T1(*)	3D TFE	1	230 × 230 × 238	7.5	291	8	Randomized	180

We use a 1.5 T Philips Ingenia scanner with a 15-channel head coil. For each experiment, the asterisk indicates the reference contrast. The “randomized” sampling pattern indicated in this table more specifically refers to variable density Cartesian randomized undersampling, while the “regular” pattern refers to classical accelerated linear filling undersampling

### Experiment 1: Robustness with respect to motion complexity

To test the robustness of the proposed motion correction scheme in terms of motion complexity, we instruct volunteer 1 to move multiple times during acquisition. With “motion complexity” we specifically refer to the number of position changes performed by the volunteer within one prospectively corrupted scan. The goal of this in-vivo study is to provide a qualitative assessment of the degradation of the reconstruction quality as a function of motion complexity.

We consider three levels of motion corruption: (i) the volunteer moves once, (ii) the volunteer moves twice, and (iii) the volunteer moves five times. The volunteer is instructed to change its head position every time it is prompted to do so, and maintain that position in between instructions. We use T2-FLAIR-weighted contrasts as corrupted scans, with T1-weighted contrast as a reference (see Table 1 for further details).

The results of this experiment are collected in “Experiment 1: Robustness test” section. Note that, in Experiment 4 (see “Experiment 4: Comparison of motion correction with and without a reference guide” section below) we use the same settings detailed in this experiment to compare the proposed algorithm with a baseline method without a reference guide.

### Experiment 2: On the choice of the reference contrast

This in-vivo experiment tests the proposed correction scheme with respect to a different combination of corrupted and reference contrast, namely a T1-weighted corrupted contrast with a T2-weighted reference contrast (see Table 1). We gather the results for this experiment in “Experiment 2: On the choice of the reference contrast” section.

For these datasets, we also test the robustness of the proposed correction mechanism with respect to the resolution of the reference contrast by artificially degrading its resolution, realized here as smoothing. The smoothing procedure is performed by low-pass filtering, where the cutoff spatial frequency *f*_max_ (in each direction) is: (i) level 0, the Nyquist frequency *f*_max_ = *f*_Nyquist_ (e.g., no smoothing applied), (ii) level 1, *f*_max_ = *f*_Nyquist_/4, (iii) level 2, *f*_max_ = *f*_Nyquist_/8. For this experiment, we prompt volunteer 2 to move five times during the acquisition.

### Experiment 3: Scanner reconstruction versus processed raw *k*-space data as input for retrospective motion correction

With the in-vivo studies presented in this section, we investigate a question related to the nature of the input data **d** (Eq. 6) required by the algorithm. Due to the formulation of the problem directly in *k*-space (by means of the NUFFT), the method assumes coil-combined data. One must then assess whether the scanner reconstruction (available in the DICOM format) is suitable for this purpose, since many different reconstruction methods are available depending on the acquisition protocol. In particular, the default reconstruction method for linear-filling patterns in *k*-space employs the SENSE framework [37], while compressed-sensing reconstruction (via the wavelet transform) is used for randomized acquisitions [38]. Note that our experimentation suggests that without the phase map of the scanner reconstruction our motion correction scheme does not perform adequately. Therefore, with “scanner reconstruction”, we will always refer to the complex-valued scanner reconstruction (comprising both the respective amplitude and phase).

In the first experiment, we asked volunteer 3 to change position once during the prospectively corrupted acquisition. We consider a corrupted T2-weighted contrast and a reference T1-weighted contrast (see Table 1). One important aspect of this experiment is related to the acquisition protocol of the T2-weighted contrast, based on a linear-filling pattern in *k*-space. In this case the corrupted data used as input for the proposed motion-correction algorithm is obtained by exporting the reconstructed volume directly from the scanner, followed by a simple Fourier transform. Note that this 3D image has been obtained by a SENSE reconstruction.

The second experiment is set up similarly to the previous one. We asked volunteer 3 to change position only once during the acquisition phase. We consider, now, a corrupted T2-FLAIR contrast with a reference T1-weighted contrast (see Table 1). The most important difference with the previous experiment, besides the type of contrast pair considered, is related to the randomized acquisition protocol. In this case, the scanner reconstruction employs a compressed-sensing reconstruction, and is not suited as input for the proposed motion-correction algorithm (see Appendix B, online resource 1). Therefore, for adequate motion correction, we must set up an intermediate step for processing the raw *k*-space data via the SENSE reconstruction.

We further discuss the results of this experiment in “Experiment 3: Scanner reconstruction versus raw *k*-space data” section.

### Experiment 4: Comparison of motion correction with and without a reference guide

The reference-guided motion-correction algorithm described in “Theory” section is compared with a standard retrospective motion-correction algorithm based on the TV regularization. We note that most retrospective motion-correction methods follow the basic mathematical framework detailed in “Theory” section (see, for example, [19] or [21]), where the main mathematical difference consists in the choice of the regularization term $g_u$, in Eq. (5). Hence, to assess the effect of the reference contrast, we adopt the same formulation described in “Theory” section with a simple TV regularization term $g_u (\mathbf{u}) = \sum \nolimits_{{\mathbf{x}}} \|\nabla {{\mathbf{u}}}|_{\mathbf{x}}\|$ (cf. Eq. 8 for the reference-guided version of TV). For the simple TV regularization term, the implementation strategy and the choice of the corresponding weights was the same as for the proposed structure-guided TV regularization (see “Optimization” section).

For the comparison with the baseline method, we use the same experimental settings in “Experiment 1: Robustness with respect to motion complexity” section. Once again, the motion artifacts are prospectively induced by prompting the volunteer to move during the scan.

## Results

In this section, we display and briefly analyze the results of the experiments presented in the previous section. We organized the peak signal-to-noise ratio (PSNR) and structural similarity index (SSIM) values of the reconstructions (with respect to a known ground truth) in several figures and tables (see Tables 3, 4, 5, 6). We compute these quality metrics both for the displayed 2D slices (values are reported directly in the figures) and for the full 3D volume of the corrupted/corrected results. As of 2D slices, we select one section per sagittal, coronal, and axial view that intersects the center of the volume. Note that both PSNR and SSIM are applied to the amplitude of the results, normalized by the highest amplitude of the ground-truth volume. For experiment 2 (varying smoothness of the reference contrast) we also compute the High Frequency Error Norm (HFEN [39]) and report it alongside the PSNR and SSIM value (Table 4).

The motion-corrected full-volume scans were analyzed by a neuroradiologist with 16 years of experience. These were generally deemed of good radiological quality. Broadly speaking, the motion-related artifacts were judged almost completely removed, and the results quite close to the ground truth. In Table 2, we organized a more detailed qualitative analysis of the 3D results, geared toward a radiological assessment of the corrected scans.

**Table 2:** Qualitative radiological analysis of the motion-corrected results shown in “Results” section. The corrected scans are radiologically similar to the ground truth

Description	Contrast (corrected)	Motion	Blurring	Additional artifacts	Other comments
*Experiment 1*					
Move once	T2-FLAIR	Completely corrected	Some blurring	n/a	Good gray/white matter differentiation
Move twice	–	Completely corrected	Some blurring	n/a	Good gray/white matter differentiation
Move five times	–	Completely corrected	Some blurring	Darker areas within the white matter	Good gray/white matter differentiation
*Experiment 2*					
Reference smoothness level 0	T1	Completely corrected	Some blurring	n/a	Good gray/white matter differentiation. Some loss of gray matter (low signal)
Reference smoothness level 1	T1	Completely corrected	Some blurring	n/a	Good gray/white matter differentiation. Some loss of gray matter (low signal)
Reference smoothness level 2	T1	Motion still present	Increased blurring	Yes	Poor gray/white matter differentiation due to motion
*Experiment 3*					
Input data from reprocessed DICOM	T2	Completely corrected	No blurring	n/a	n/a
Raw input data	FLAIR	Completely corrected	Some blurring	n/a	Good gray/white matter differentiation

### Experiment 1: Robustness test

**Fig. 1:**
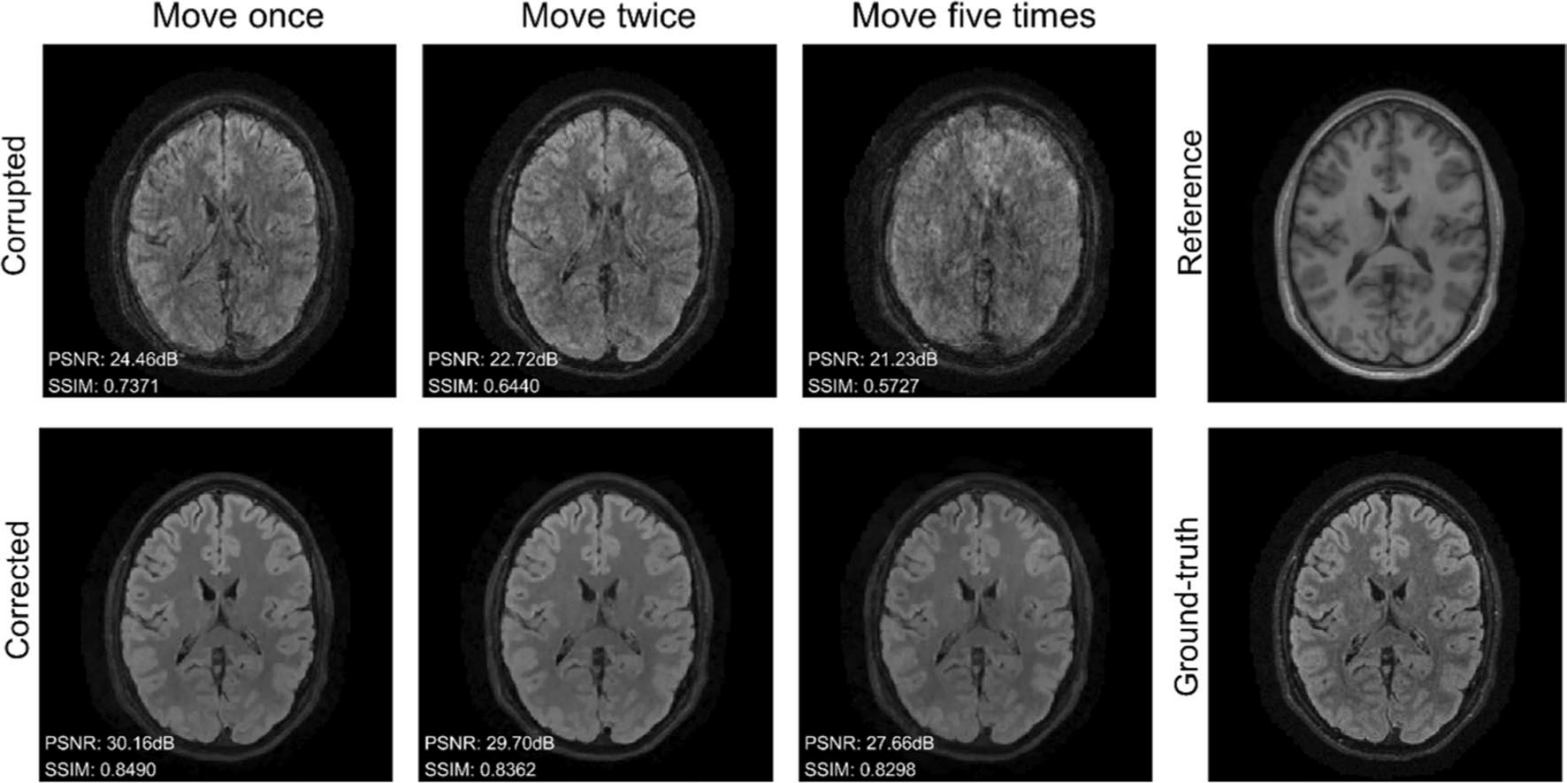
Summary of the reconstruction results for Experiment 1 (axial view). The volunteer is instructed to move a variable number of times during the scan to test the robustness of the proposed correction scheme with respect to the motion complexity. The corrupted images are increasingly affected by motion artifacts, however, only modest decrease in reconstruction quality can be observed for the corrected images

**Fig. 2:**
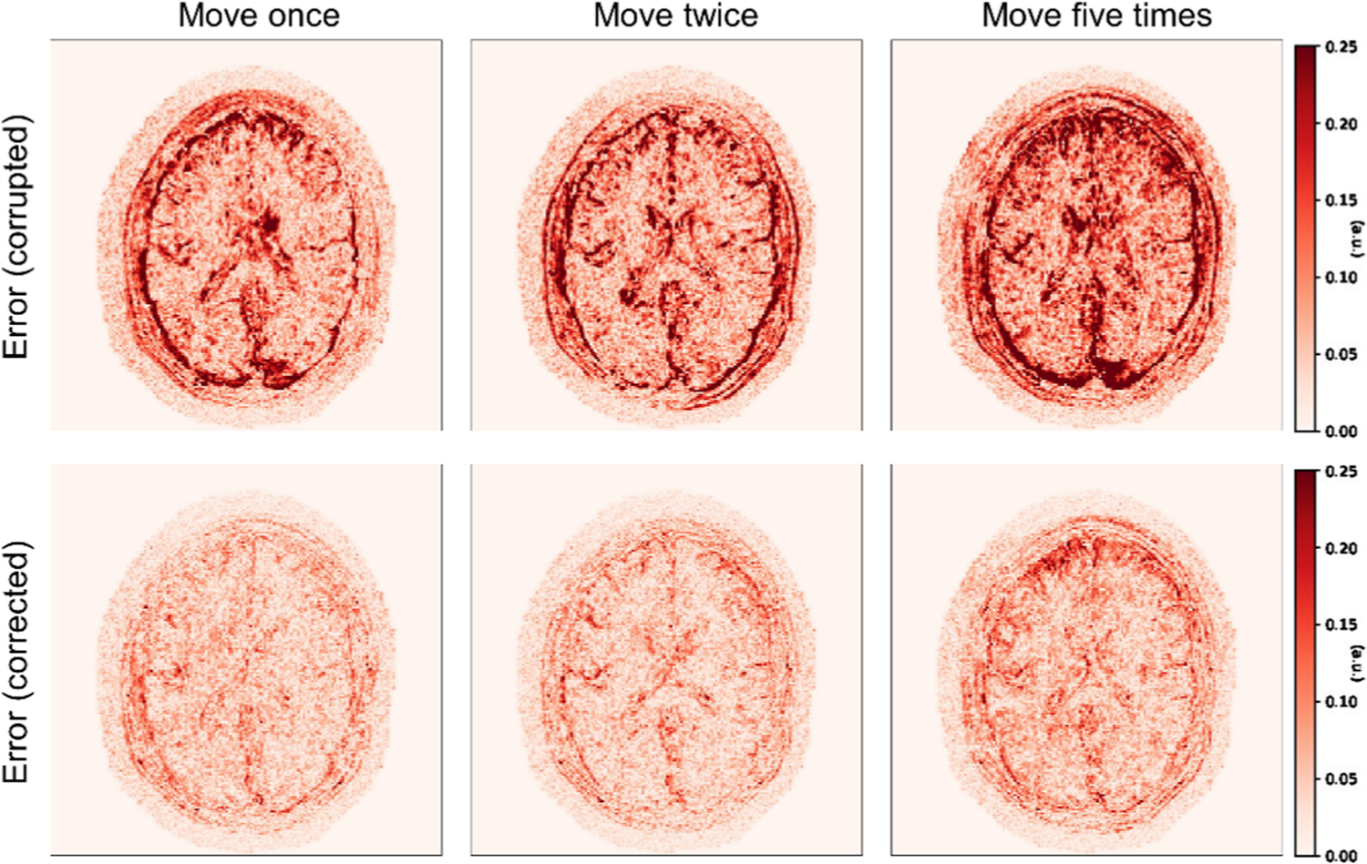
Normalized error maps with respect to the ground-truth for the results of Experiment 1 shown in Fig. 1 (the normalization constant is the maximum amplitude of the ground-truth)

**Fig. 3:**
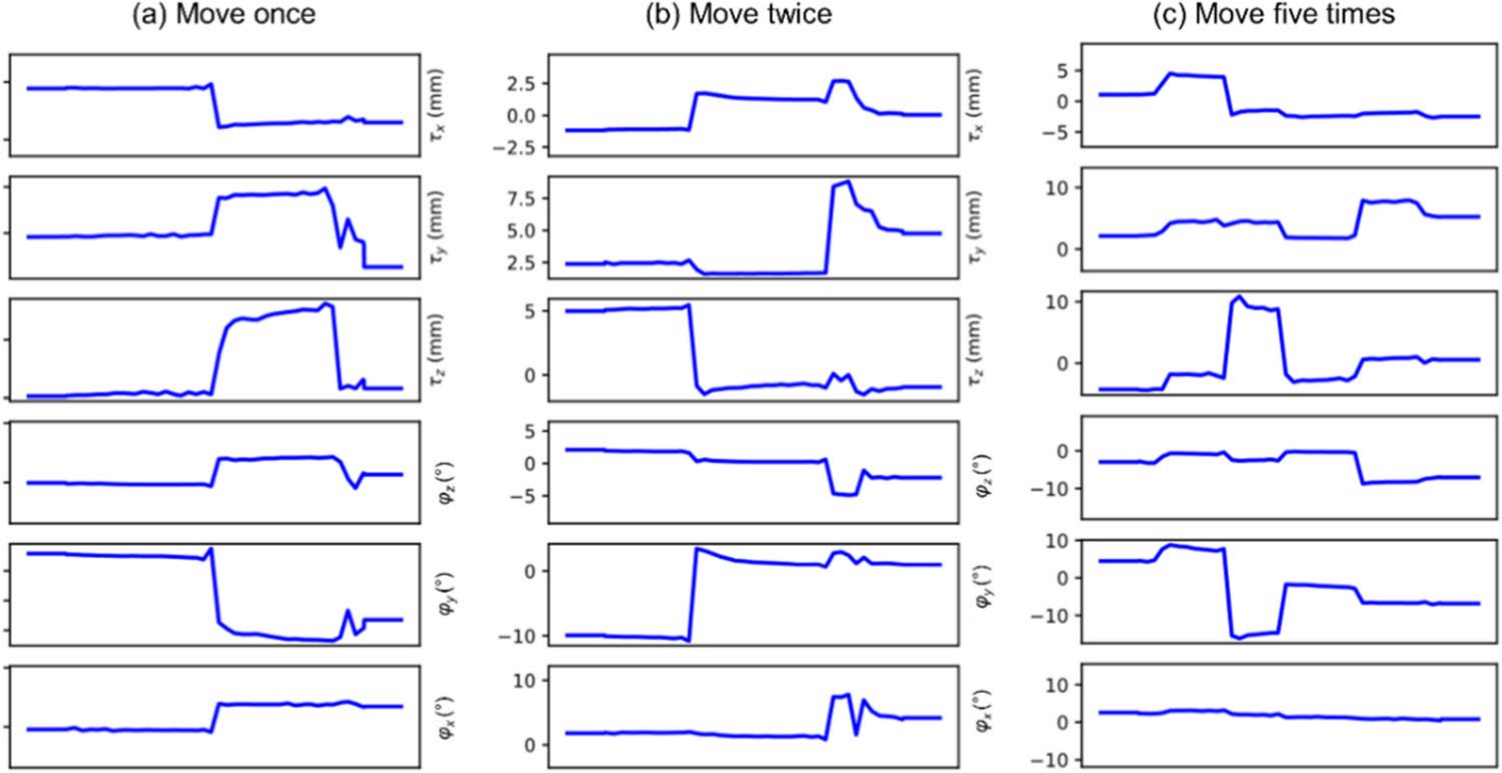
Estimated rigid motion parameters for Experiment 1 (Fig. 1). The volunteer was asked to move several times during the scan: **a** once, **b** twice, and **c** five times

We gather the results for the robustness test, described in “Experiment 1: Robustness with respect to motion complexity” section (volunteer 1), in Fig. 1 (see also Appendix A, online resource 1, Figs. S.1–S.3), where we juxtaposed the corrected images with varying degrees of corruption. We observe that the proposed method consistently ameliorates the corrupted scan. For this set of specific motion tests, the quality indexes based on PSNR and SSIM show only a modest decrease in correction quality as a function of motion complexity (see Table 3). To better assess the influence of the motion complexity of the results, we included a comparison of the corrupted and corrected error maps in Fig. 2. Additionally, the recovered motion parameter trajectories are presented in Fig. 3. The step-wise behavior of the parameters is in good qualitative accordance with the instructions given to the volunteer.

**Table 3:** Summary of the motion-correction results of Experiment 1 in terms of PSNR and SSIM

Description	PSNR (dB) ↑		SSIM ↑	
	Corrupted	Corrected	Corrupted	Corrected
Move once	27.22	31.64	0.8266	0.8755
Move twice	26.15	31.43	0.7946	0.8690
Move five times	24.52	29.35	0.7326	0.8645

### Experiment 2: On the choice of the reference contrast

**Fig. 4:**
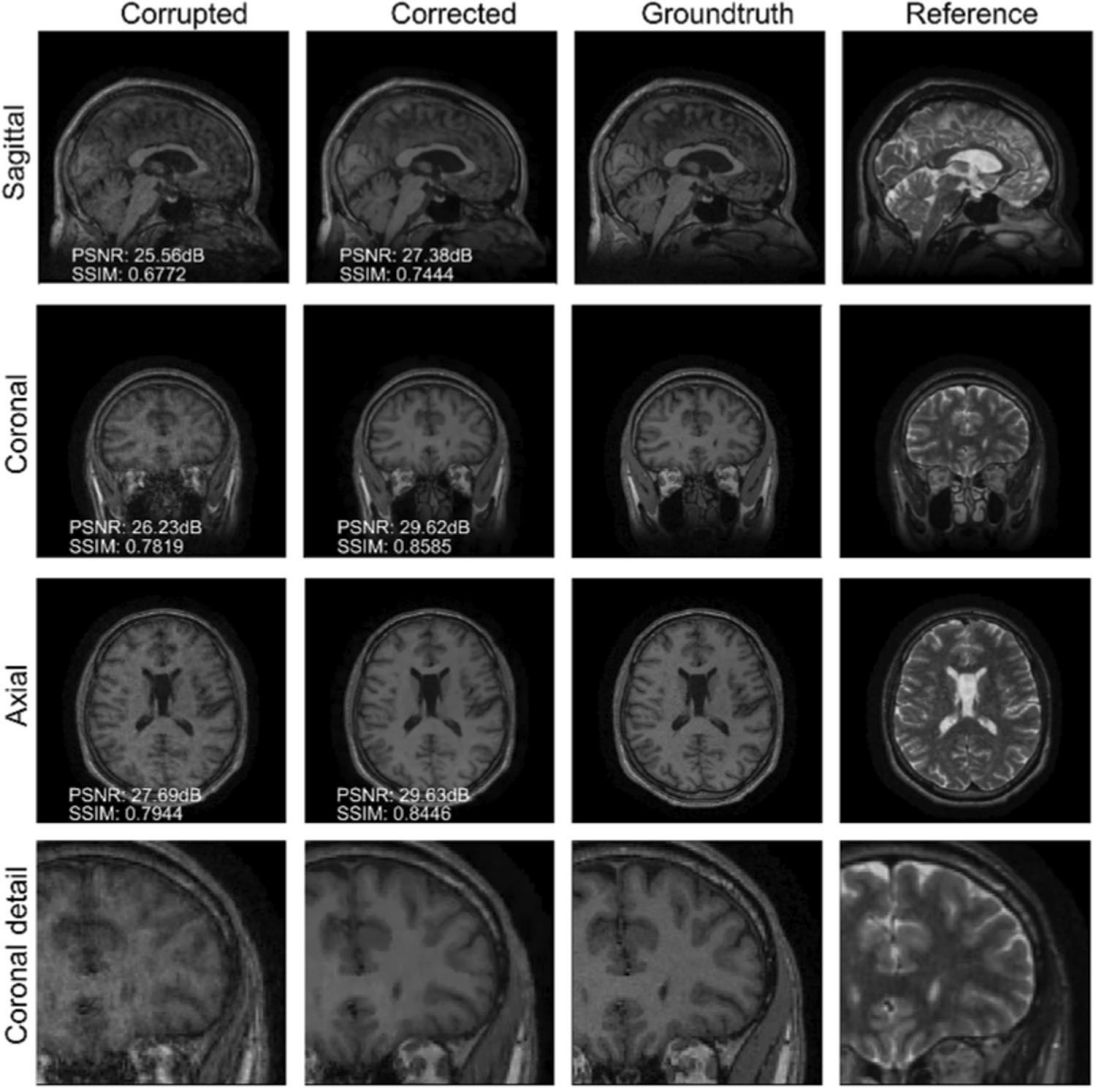
Reconstruction results for Experiment 2. The reference scan is not smoothed

The results are shown in Fig. 4 (see also Appendix A, online resource 1, Figs. S.4–S.7). Contrary to the experiments detailed in the previous section, we are now considering a T2-weighted reference contrast to guide the correction of a T1-weighted corrupted contrast. The quality of the correction indicates that the proposed technique is relatively robust in terms of reference contrast, although the corrected scans degrade noticeably when the reference image is heavily smoothed (i.e., much lower resolution than the target image), as it can be assessed from the error maps in Fig. S.7 and the radiological comments in Table 2. See also Table 4 for more detailed PSNR, SSIM and HFEN quality metrics. We also note that the lack of gray/white contrast in the reference scan might negatively bias the correction (see the highlighted details in Fig. 4).

**Table 4:** Summary of the motion-correction results of Experiment 2 in terms of PSNR, SSIM and HFEN

Description	PSNR (dB) ↑		SSIM ↑		HFEN ↓	
	Corrupted	Corrected	Corrupted	Corrected	Corrupted	Corrected
Reference smoothness level 0	28.28	30.24	0.8408	0.8697	0.5727	0.3612
Reference smoothness level 1	–	30.09	–	0.8659	–	0.3839
Reference smoothness level 2	–	28.98	–	0.8465	–	0.4958

### Experiment 3: Scanner reconstruction versus raw *k*-space data

**Fig. 5:**
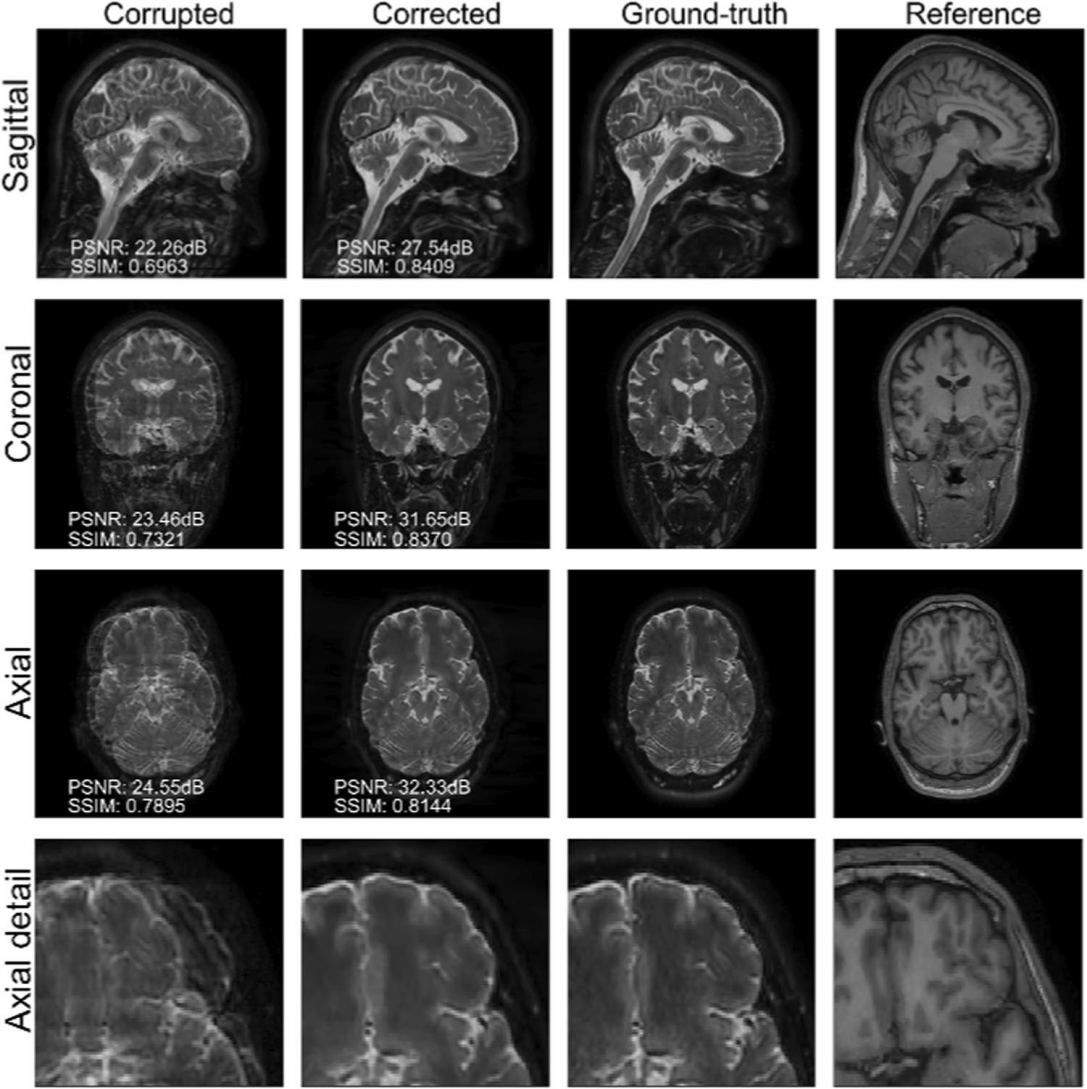
Reconstruction results for Experiment 3. The volunteer is instructed to move once, halfway through the scan. In this case, the input data for the correction algorithm are directly extracted from the scanner reconstruction in DICOM format (comprising both amplitude and phase). The acquisition scheme for the corrupted contrast follows a linear filling pattern in *k*-space. The proposed method successfully removes the motion artifacts because the scanner reconstruction is obtained through a conventional SENSE reconstruction (cf. Fig. 6)

**Fig. 6:**
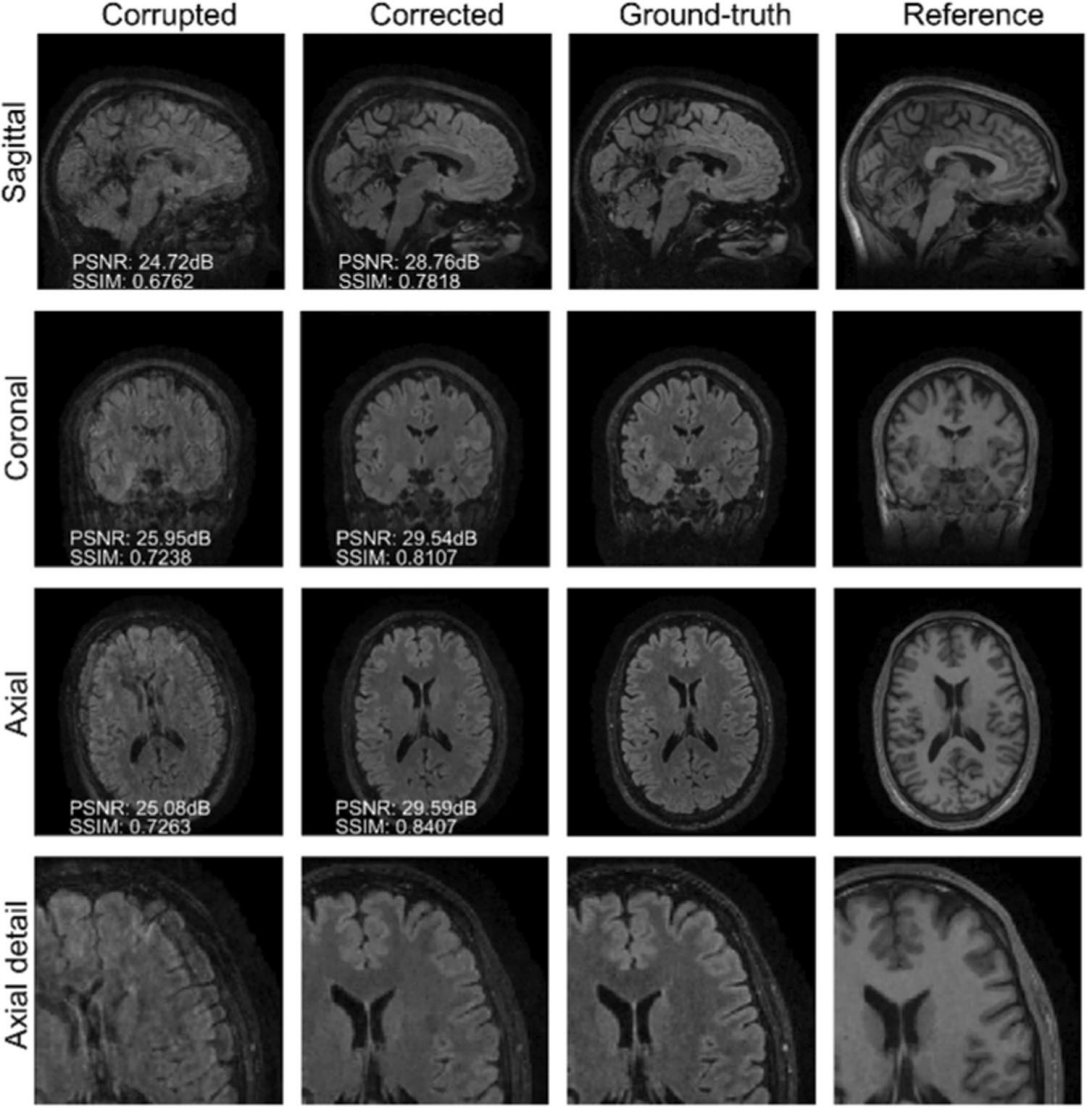
Reconstruction results for Experiment 3. The volunteer is instructed to move once, halfway through the scan. Unlike in Fig. 5, the input data for the correction algorithm are obtained via a preliminary SENSE reconstruction of the raw *k*-space data. When the scanner reconstruction is directly processed to input data via the Fourier transform, the motion correction is highly defective (cf. Fig. S.8 in Appendix B, online resource 1)

The results of the two experiments described in “Experiment 3: Scanner reconstruction versus processed raw *k*-space data as input for retrospective motion correction” section are depicted in Figs. 5 and 6. The main difference between the two experiments is related to the input data for the proposed motion-correction algorithm. We refer to Table 5 for detailed PSNR/SSIM quality metrics.

**Table 5:** Summary of the motion-correction results of Experiment 3 in terms of PSNR and SSIM

Description	PSNR (dB) ↑		SSIM ↑	
	Corrupted	Corrected	Corrupted	Corrected
Input data from reprocessed DICOM	25.95	32.48	0.8392	0.8573
Raw data input	27.84	31.53	0.8080	0.8643

In the first experiment, the corrupted contrast has been acquired with a protocol based on a linear filling pattern in *k*-space. Note that, in this particular case, the scanner reconstruction implements the SENSE method. We then extracted the DICOM of both amplitude and phase produced by the scanner, and reprocessed the complex image to form *k*-space data via the Fourier transform, to be used as input for the Algorithm 1. The proposed scheme is able to successfully remove the motion artifacts in Fig. 5.

In the case of randomized sampling, the scanner reconstruction is not adequate as input data for the proposed motion-correction algorithm, because it employs a compressed-sensing algorithm. We speculate that compressed-sensing reconstructions degrade the information contained in the corrupted volume, and the corrected contrast cannot be effectively recovered by simply removing rigid-motion artifacts (we defer the degraded results when using scanner reconstruction data in Appendix B, online resource 1). However, when the input data are obtained by directly processing the raw *k*-space data via the SENSE reconstruction, the motion-correction scheme is able to successfully remove the motion artifacts (Fig. 6).

### Experiment 4: Comparison of motion correction with and without a reference guide

**Fig. 7:**
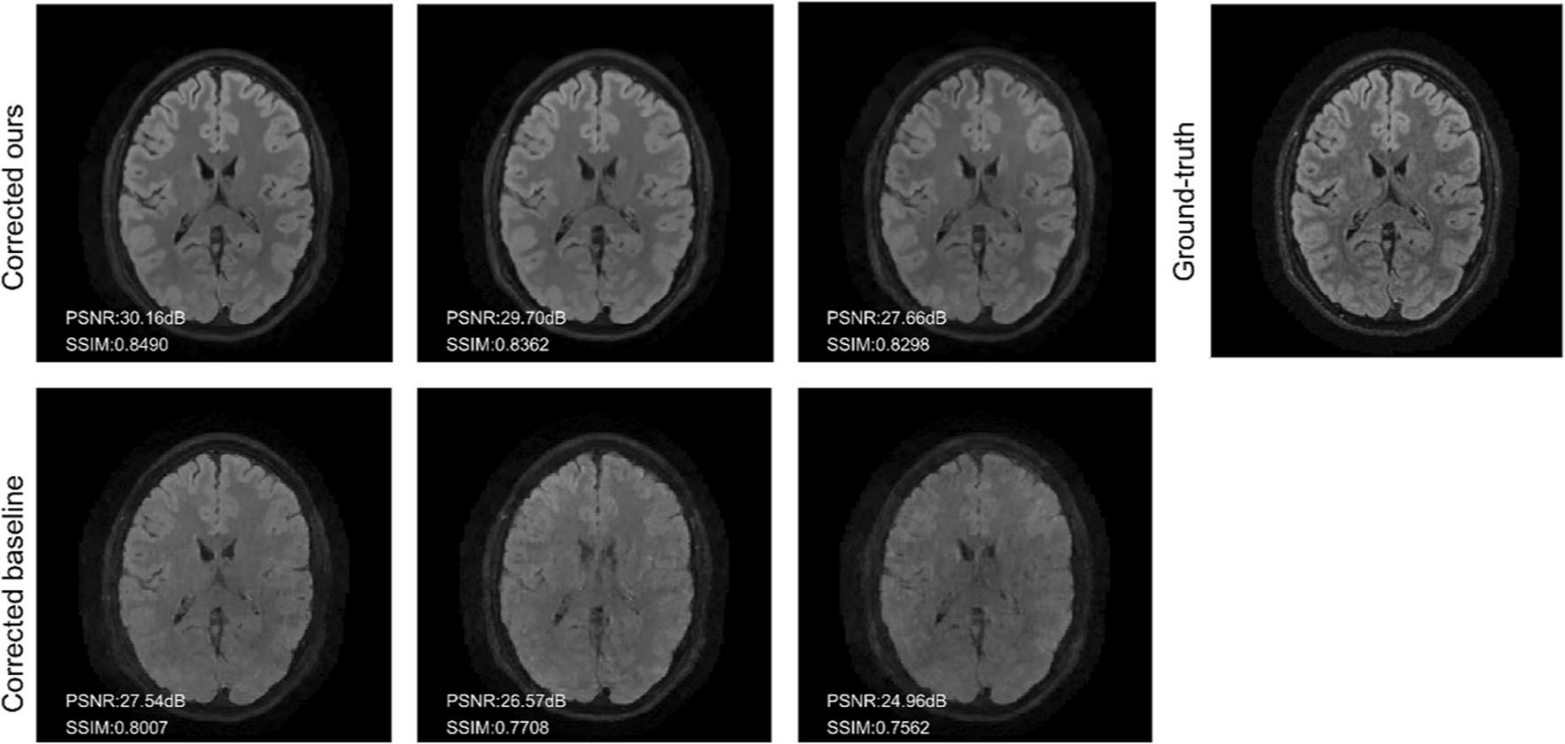
Experiment 4. Comparison of the reconstruction results for Experiment 1 with a reference-guided (ours) and a baseline motion-correction method (e.g., not guided by a reference contrast). The volunteer is instructed to move a variable number of times during the scan to test the robustness of the proposed correction schemes with respect the motion complexity. The corrupted images are increasingly affected by motion artifacts. The decrease in reconstruction quality for the baseline method is substantially more pronounced than the results obtained with our reference-guided correction

**Fig. 8:**
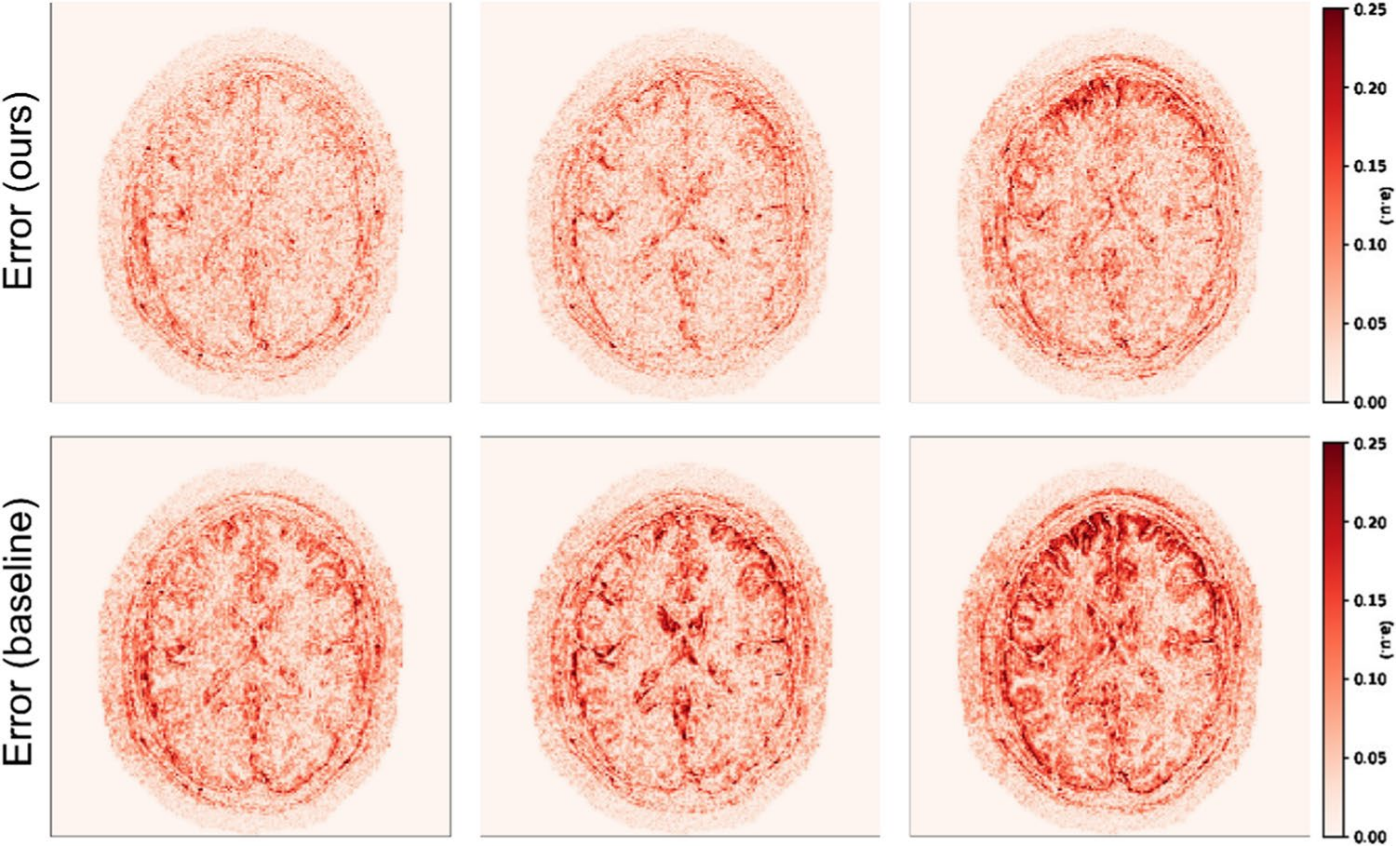
Experiment 4. Normalized error maps with respect to the ground-truth for the results of Experiment 1 shown in Fig. 7 (the normalization constant is the maximum amplitude of the ground-truth)

The results are summarized in Figs. 7, 8 and Table 6.

**Table 6:** Experiment 4. Comparison of the motion-correction results for the baseline and reference-guided methods in terms of PSNR and SSIM

Description	PSNR (dB) ↑			SSIM ↑		
	Corrupted	Corrected		Corrupted	Corrected	
		Ours	Baseline		Ours	Baseline
Move once	27.22	**31.64**	29.51	0.8266	**0.8755**	0.8497
Move twice	26.15	**31.43**	28.68	0.7946	**0.8690**	0.8309
Move five times	24.52	**29.35**	26.92	0.7326	**0.8645**	0.8192

Bold fonts indicate the values with the best performance

We note that the difference in performance between the reference-guided and blind motion correction is even more pronounced in this example than what was previously shown in [29] (which was limited to 2D synthetic data). It is also worth noting that, in our experience, the results for blind motion correction depend more sensibly on the choice of the hyper-parameters in Eq. (5) than the proposed reference-based version.

## Discussion

Reference-guided TV regularization substantially improves the motion correction quality, both visually and in terms of quality metrics based on PSNR and SSIM, when compared to basic reconstruction methods without motion correction. As reported in Table 2, most corrected images contain residual blurring; however, this degree of blurring does not hamper the radiological inspection of the images. The comparison is also substantially favorable with standard “blind” motion correction techniques, for example, based on conventional regularization such as TV, which do not employ a reference to guide the correction. In fact, for randomized sampling patterns that are now common in the clinical practice, we verified that blind retrospective techniques are wholly inadequate for motion correction of radiological quality (cf. the comparison in Figs. 7 and 8 and Table 6).

Our experimentation based on volunteer data aimed at assessing the robustness of the correction quality with respect to motion artifacts of increasing complexity. In this study, we equated this complexity to the number of volunteer changes of pose during the acquisition phase. Clearly, this does not fully describe the complexity of motion encountered in practice in the clinic, but it only constitutes a preliminary step in that direction. Nevertheless, the results described in “Experiment 1: Robustness test” section support the indication that the retrospective motion correction based on a reference contrast is quite robust in terms of reconstruction quality, with only minor degradations in terms of contrast and resolution.

The flexibility of the proposed motion-correction method is demonstrated with different combinations of motion-corrupted and reference contrasts (“Experiment 2: On the choice of the reference contrast” section). Our experience suggests that one important factor in assessing the effectiveness of the reference contrast as a guide for motion correction lies in the similarity of the *k*-space distribution of the two contrasts. Good reconstruction quality can be expected when the reference contrast has similar or higher frequency content when compared to the corrupted contrast, regardless of the type of contrast considered. The results in Fig. S.6 show that the correction will gradually lose its effectiveness with increasing reference smoothness. More generally, our experiments highlight that the imprint of the reference scan has important consequences in the quality of the reconstructed results. For example, in Fig. S.5, the low definition of white matter/gray matter contrast in the reference image is reflected on the motion-corrected results. Based on these observations only, it is difficult to predict the performance of the method with inconsistent tissue boundaries (contrast) between reference and target images such as it may occur in (large) pathologies. Future clinical validation will be needed to assess the performance of the method in these cases.

A significant part of our experimentation was devoted to assess whether the scanner reconstruction (available as DICOM format) can be directly reprocessed via the Fourier transform and subsequently used as input data for the proposed correction method (“Experiment 3: Scanner reconstruction versus raw *k*-space data” section). We established that the scanner reconstruction is not suitable for this purpose when it is obtained via compressed-sensing algorithms (Appendix B, online resource 1, Fig. S.8), which is the case for randomized sampling on the 1.5 T Philips Ingenia scanner utilized in this work. In this case, we must reprocess the raw *k*-space data and perform an intermediate SENSE reconstruction for effective motion correction. Although we did not apply our method to data reconstructed with deep-learning-based techniques [40], we can expect that the highly non-linear aspect of the framework would render the reconstruction at least as difficult as when working with the CS-based reconstructed data. Also in this case, the solution should be to process directly the raw-*k*-space data.

Our method circumvents the need for reconstructions based on separate multi-coil datasets. The advantage is mostly computationally, since the number of NUFFT operations is drastically reduced when all the information is compressed into one (virtual) channel. We are aware that the practical approach adopted in the experiments in dealing with parallel imaging is not perfectly consistent with the motion corruption effects. The main qualitative argument used to justify our approach consists in these assumptions: (i) the coil sensitivity maps are smooth in space (hence, the associated convolutions in *k*-space are local), (ii) the motion trajectories are mostly smooth in time. If one accepts the aforementioned approximation (and looking at the results in this work there seems to be no reason to reject it) our approach is much superior in terms of computational complexity with respect to conventional implementations (as in [21]). The conventional, multi-channel implementation would need a multiplication with the coil maps at each motion change; therefore, at each time point a FFT would be needed which would amount to ${\text{O}}(n_t {*}n_x {*}\log n_x )$ where $n_t { }$ is the number of time steps (equivalent to the phase encoding steps, which in 3D is ${\text{O}}(n_x^2 ))$, while $n_x$ is the grid size (for every “time” point, one needs to perform rigid motion + FFT). Also, in [21] piecewise constant motion is considered to reduce $n_t$. Our single-channel approach, by contrast, is only ${\text{O}}(n_x {*}\log n_x )$ (i.e., a single NUFFT evaluation) because we do not need the multiplication step with the coil maps. In conclusion, our approach reduces the computational complexity by ${\text{O}}(n_x^2 )$ factor.

The performance comparison between the proposed sTV regularization and the conventional TV regularization (experiment 4) shows the merit of the reference-guided strategy in our single-channel implementation. Considering that all data from this study is under-sampled, one might wonder what would happen if a multi-channel reconstruction were implemented. This is difficult to predict. Our previous study and comparison based on fully sampled, motion-corrupted 2D data [29] suggest that, even when spatial encoding is totally resolved, motion is much better resolved with sTV than TV. On the other hand, the seminal work by Ehrhardt et al. [30] considers image reconstruction from motion-free, under-sampled data and points out at the superiority of sTV with respect to TV alone. This seems to suggest that the gap between sTV and TV might narrow when spatially encoding is properly taken into account, for instance by means of multi-channel signal models. Note, however, that the joint image/motion reconstruction problem considered in our work involves also the reconstruction of the motion parameters and as such introduces additional complexity in the inverse problem. Consequently, applying the conclusions from [30] (where no motion parameters are reconstructed) to our work could be misleading. In conclusion, we believe that only a thorough comparison study with multi-channel implementations of under-sampled and motion-corrupted scans can indicate how much the gap between TV and sTV would narrow. Such a study goes beyond the scope of this work. Nonetheless, the findings presented here offer substantial evidence of the practical benefits and efficacy of our motion-correction methodology within a single-channel framework.

The computational times of the motion correction are, generally speaking, problem dependent, since complex motion artifacts require an increasing number of iterations as a function of motion complexity (“Optimization” section). The examples illustrated in this study, where a fixed number of iterations was considered irrespectively of motion complexity, are completed within 1 h 30 min for 3D images of approximately 256 × 256 × 256 voxels. The current CPU implementation was run on a consumer grade laptop with the following processor specifications: Intel Core i7-10750H CPU@2.60 GHz × 12. An effective implementation in a clinical scenario for on-line reconstructions will likely require GPUs.

Considering data storage issues, it is clear that our method would require more room to store also the phase data. In case of DICOM inputs, this would mean a doubling of the data. But if all raw multi-channel complex *k*-space data have to be stored, additional memory space might need to be created in the imaging server. However, *k*-space data can be deleted as soon as the proposed reconstruction algorithm is terminated; only the corrected (magnitude) images need to be stored and memory can be freed up again. We realize that our method can be regularly applied only when the option for storage complex data is activated; this is relatively straightforward for future exams but data from past studies rarely include phase information, thus cannot be processed by our pipeline.

The basic assumption of the proposed retrospective correction method is related to the availability of a motion-free 3D reference contrast. From the experience in our clinical practice and from literature [1] we can conclude that the cases in which all sequences of an exam are corrupted are extremely rare. In most exams, early sequences are usually motion free and can thus be used as reference for the correction of later acquisitions. We note that the reference contrast may also come from previous MR sessions, with the caveats highlighted by the results in “Experiment 2: On the choice of the reference contrast” section. In this particular case, the bias introduced by the structural prior may have an adverse effect in case of an evolving pathology. However, when structural changes involve a limited pathological region, the adverse bias could be mitigated by masking the affected zone, although this would require substantial manual interventions in the reconstruction process. The performance of the proposed method in this scenario is left to future clinical validation.

Note that the motion-free reference can be exploited differently than the reference-guided TV regularization introduced in [30], and adopted in this work. For example, one may consider several competing techniques advanced for multi-contrast MRI, such as Bayesian compressed sensing [41], group sparsity [42], reference-based MRI [43], or multi-contrast graph-based sparsity [44, 45]. The method here presented is limited to rigid motion. Indeed, some decrease in correction quality is noticeable in Fig. 5 in the neck region (which is not supposed to behave rigidly). However, our technique may be extended to non-rigid motion and, hence, different body regions other than the brain (see, for example, [46]). A major challenge for such extension is a computationally effective parameterization of the motion effects, and the resulting ill-posedness of the inverse problem.

Note that a significant computational advantage of rigid motion over non-rigid motion is related to the direct implementation of the rigid motion in *k*-space, via Eq. (3), which results in a data model that requires a single NUFFT evaluation, regardless of the number of time samples considered. Other interesting extensions of the method are related to the integration of specialized motion-resilient acquisition patterns, e.g., as described in [21].

## Conclusions

We assessed the performance of the proposed retrospective motion correction method based on a reference contrast not affected by motion artifacts. The current prospective in-vivo study targets 3D clinical protocols conventionally used in brain imaging. The method is tested with several degrees of motion artifacts, by instructing the volunteers to change position during the scan multiple times. While we observe that the corrupted images are severely degraded as a function of motion complexity, the corrected images are generally robustly estimated. We also verified that the proposed technique is quite flexible with respect to the choice of the reference contrast, as long as the frequency content of the reference and target contrasts is comparable. Further assessment of the proposed method will be devoted to patient data.

## Supplementary Information

Below is the link to the electronic supplementary material.

Supplementary file1 (DOCX 3930 kb)

## Data Availability

The 3D results of the experiment described in “Methods”–“Results” sections are freely available online in the DICOM format at the following link: https://github.com/grizzuti/ReferenceGuidedMotionCorrection_Supplementary_DICOM.

## Footnotes

1 We have informed written consent from the volunteers. The experiments were approved by the ethical review board of the UMC Utrecht.
