# Supplementary material for: Towards retrospective motion correction and reconstruction for clinical 3D brain MRI protocols with a reference contrast
Source: MAGMA. 2024 May 17;37(5):807–23. doi: 10.1007/s10334-024-01161-y (PMC11452448; doi:10.1007/s10334-024-01161-y)
Supplement: Supplementary file 1 — Supplementary file1 (DOCX 3930 kb) [file 10334_2024_1161_MOESM1_ESM.docx]

Magnetic Resonance Materials in Physics, Biology and Medicine

*Supplementary material to*

Towards retrospective motion correction and reconstruction for clinical 3D brain MRI protocols with a reference contrast

Gabrio Rizzuti^a,b^, Tim Schakel^b^, Niek R. F. Huttinga^b^, Jan Willem Dankbaar^b^, Tristan van Leeuwen^a,c^, Alessandro Sbrizzi^b,*^

^a^Utrecht University, ^b^Universitair Medisch Centrum Utrecht, ^c^Centrum Wiskunde & Informatica Amsterdam

* Corresponding author. Email address: [a.sbrizzi@umcutrecht.nl](mailto:a.sbrizzi@umcutrecht.nl) (Alessandro Sbrizzi)

**Appendix A. Additional results**

In this section, we provide a more detailed review of the results of Experiment 1 (Sections 3.1, 4.1) and Experiment 2 (Sections 3.2, 4.2), which aim at testing the robustness of the proposed motion-correction algorithm with respect to motion severity and reference quality. Here, we additionally provide a comparison of sagittal, coronal, and axial views. The additional results can be found in Figures S.1–S.3 for Experiment 1, and Figures S.4–S.6 for Experiment 2.

**Appendix B. Inadequate motion correction with scanner reconstruction as input data**

As anticipated in Section 3.3, directly using the scanner reconstruction (extracted as DICOM files of both the amplitude and phase of the reconstruction) as input data for the proposed motion correction scheme may degrade the performance when compressed-sensing reconstruction tools have been employed in the reconstruction process. To motivate this conclusion, we setup an experiment with the same setting as described in the second experiment in Section 3.3, the only difference being in how the input data is generated. In this case, the input data consist of the Fourier transform of the extracted scanner reconstruction. The related suboptimal correction is quite evident when comparing Figure S.8 with Figure 6.


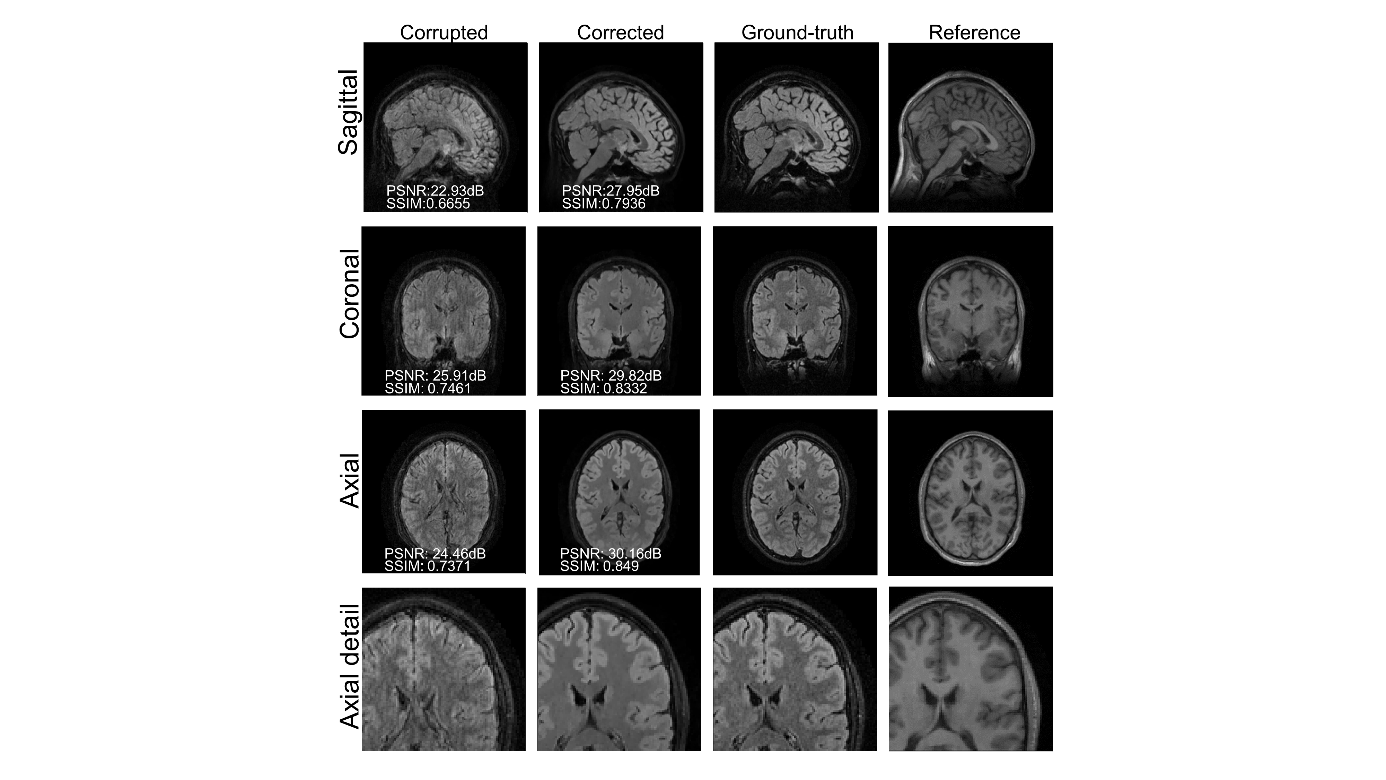


**Fig. S.1** Reconstruction results for Experiment 1. The volunteer is instructed to move once during the scan.

**
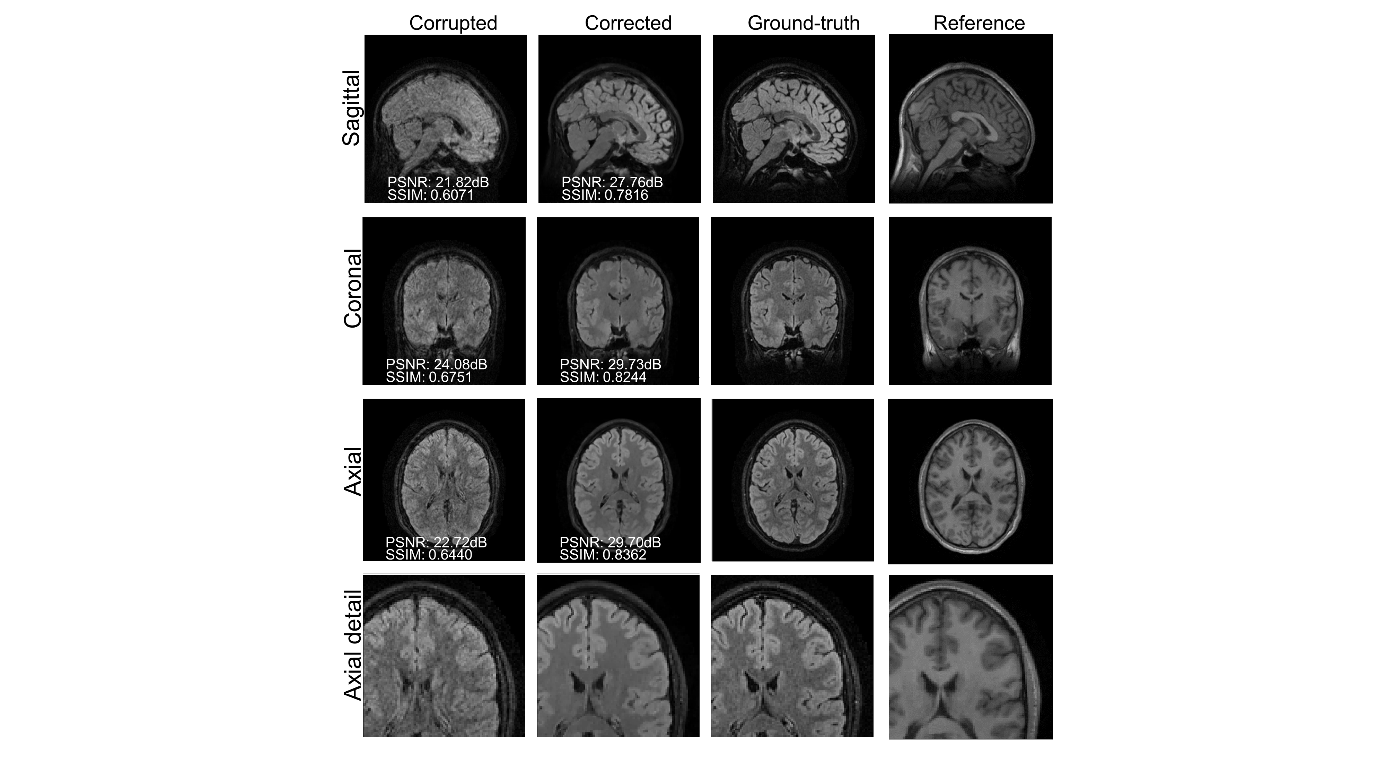
Fig. S.2** Reconstruction results for Experiment 1. The volunteer is instructed to move twice during the scan.

**
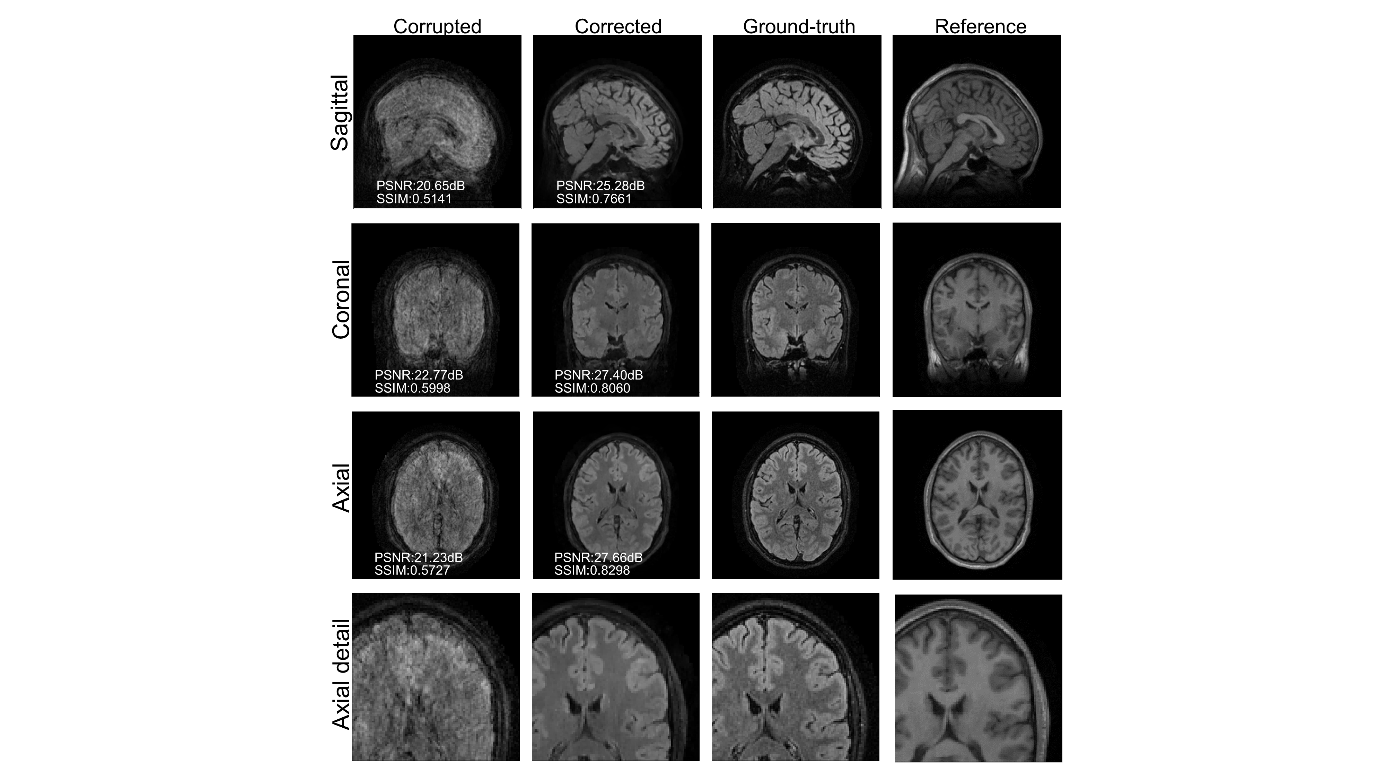
Fig. S.3** Reconstruction results for Experiment 1. The volunteer is instructed to move five times during the scan.


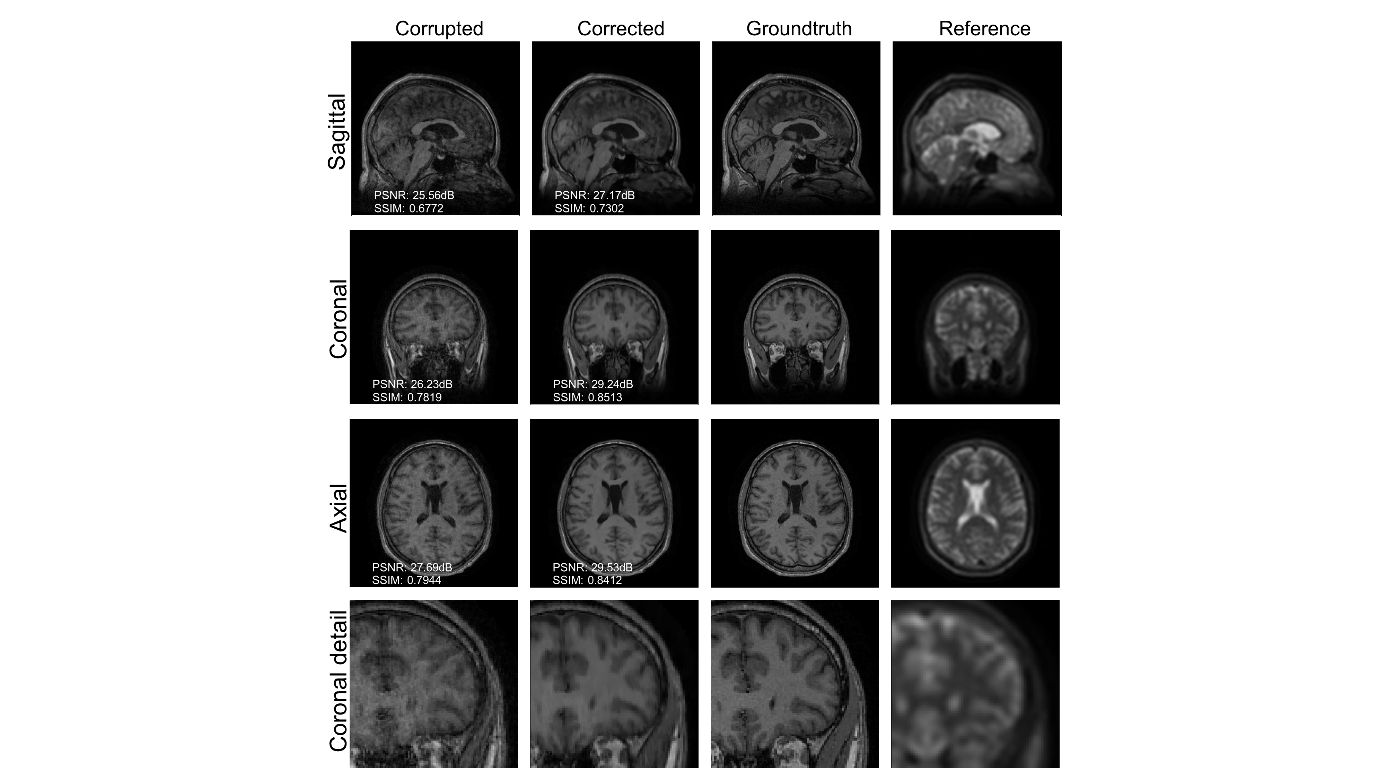
 **Fig. S.4** Reconstruction results for Experiment 2. The reference scan is smoothed via a low-pass filter such that the spatial frequencies above *f*_Nyquist_/4 are dampened.


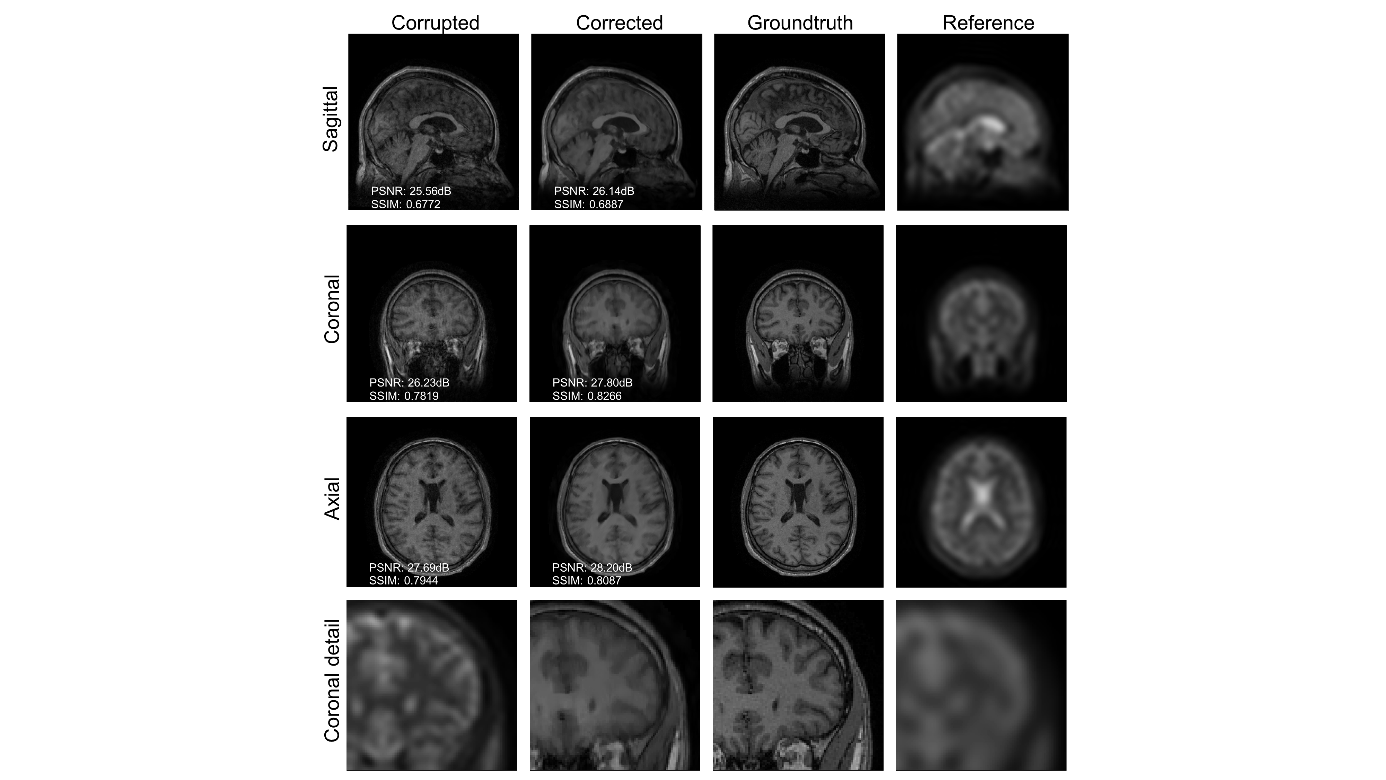
 **Fig. S.5** Reconstruction results for Experiment 2. The reference scan is smoothed via a low-pass filter such that the spatial frequencies above *f*_Nyquist_/8 are dampened.


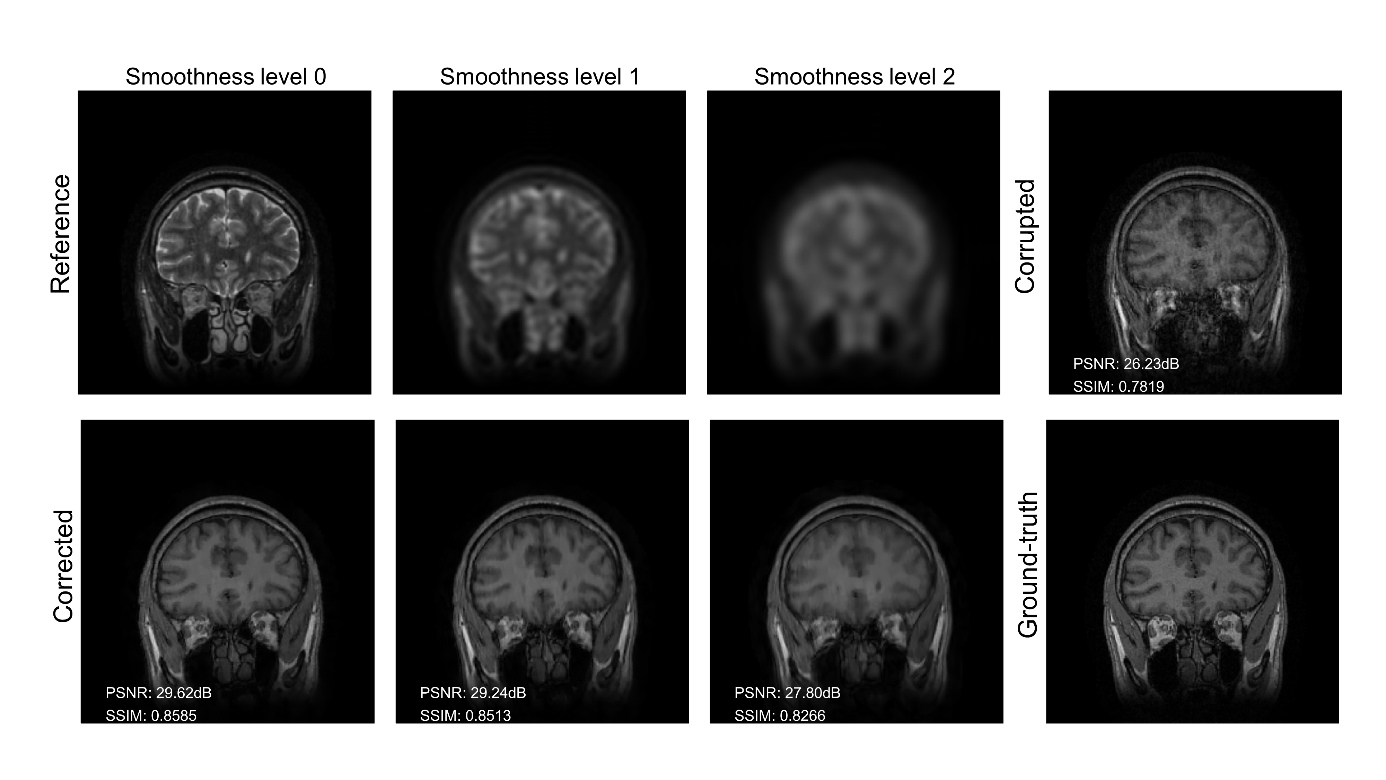
**Fig. S.6** Summary of the reconstruction results for Experiment 2 (coronal view). The volunteer is instructed to move five times during the scan. The proposed correction scheme can be successful in removing motion artifacts with several choices of corrupted/reference contrast combinations, and is relatively stable with respect to the smoothness level (i.e. acquired resolution) of the reference contrast.


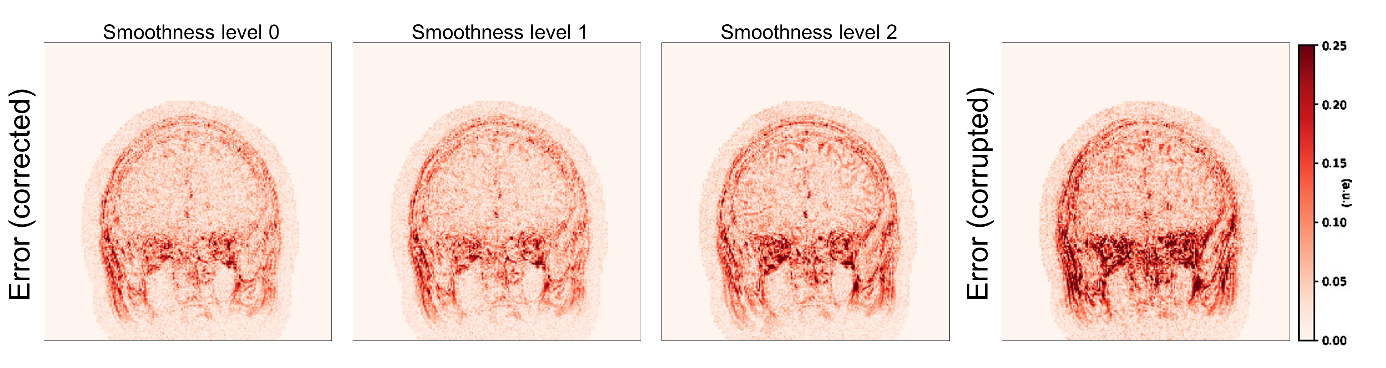


**Fig. S.7** Normalized error maps with respect to the ground-truth for the results of Experiment 2 (the normalization constant is the maximum amplitude of the ground-truth).


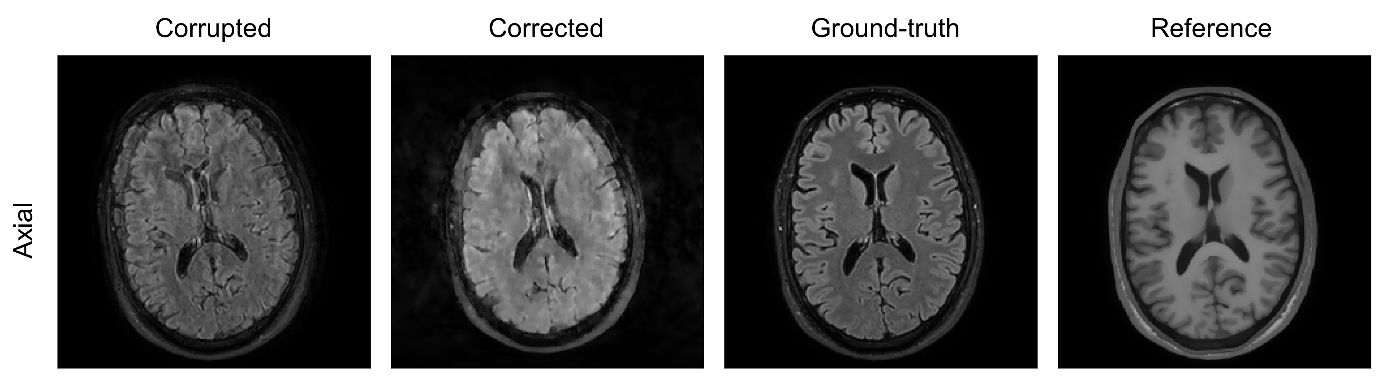


**Fig. S.8** Reconstruction results for Experiment 3. The volunteer is instructed to move once, halfway through the scan. In this experiment, the proposed motion correction scheme processes the scanner reconstruction directly. Since the reconstruction algorithm implemented in the scanner destroys the coherence of the rigid motion artifact, the proposed method cannot properly recover the correct reconstruction by simply estimating the motion parameters. With contrasts obtained by randomized acquisitions, we advise to use raw k-space data instead (cf. Figure 6).
